# Simulation Model for Hardware-in-the-Loop Tests of the ILR-33 AMBER Rocket Control System

**DOI:** 10.3390/s25134083

**Published:** 2025-06-30

**Authors:** Dawid Cieśliński, Rafał Dziczkaniec, Jan Kierski, Cezary Szczepański, Michał Welcer

**Affiliations:** Łukasiewicz Research Network–Institute of Aviation, Aleja Krakowska 110/114, 02-256 Warsaw, Poland; dawid.cieslinski@ilot.lukasiewicz.gov.pl (D.C.); rafal.dziczkaniec@ilot.lukasiewicz.gov.pl (R.D.); jan.kierski@ilot.lukasiewicz.gov.pl (J.K.); cezary.szczepanski@ilot.lukasiewicz.gov.pl (C.S.)

**Keywords:** suborbital rocket, Hardware-in-the-Loop, control system, flight dynamics, real-time, simulation, aerodynamics

## Abstract

In this paper, an advanced flight simulation model of the ILR-33 AMBER rocket is shown. The model is designed for Hardware-in-the-Loop (HiL) tests of the rocket control system. It permits us to simulate flight dynamics in a 6DOF environment, with consideration of the variable thrust, mass-inertia, and aerodynamics. It reproduces key functionalities of on-board computer and sensors and allows us to reproduce multiple mission scenarios. Simplifying assumptions concerning the environment and coordinate systems were made, reducing calculation costs while preserving key functionalities of the simulation. The control system consists of four movable canards, actuators, and motion controllers. The process of integration between the simulation model and hardware using a real-time computer is shown. Efficient communication between those elements was developed and tested in simulated flight conditions. In the final part, relevant control system HiL tests were presented. An extensive comparison between unguided and guided flight trajectories was performed. The impact of the control system operation on all analyzed parameters is clearly demonstrated. The results confirmed the usefulness of the simulation model for the task it was developed for. The potential of the HiL method in the design of complex control systems for suborbital rockets is proven.

## 1. Introduction

In general, it is considered and recommended that each system, once designed and built and before being put into service, should be subjected to a verification process that demonstrates its functional capability and operational safety [1]. This also applies to systems intended for use on-board suborbital rockets [2,3]. This kind of verification can be performed in several ways, for example, by correlation between mathematical models and bench tests, by performance tests of black-box systems, or through flight tests as a payload [4,5].

However, conducting prototype tests in an operational environment, i.e., during flight tests, is often associated with high costs. Extensive ground infrastructure and compliance with stringent safety standards are also required for these types of tests. For this reason, efforts are made to minimize the number of necessary flight tests and conduct them only at the final qualification stage of the entire launch system [6].

On the other hand, standard verification methods at the component level, such as calculations, analysis, and bench tests, are not fully representative in reference to flight conditions and thereby do not provide an adequate level of confidence. This is especially important in the case of safety-critical systems such as seekers, navigation units, on-board avionics, and actuators (valves, mechanisms, and initiators). For these types of components, an HiL method was developed to simulate their operation coupled with a relevant virtual environment.

To perform these types of tests, a high-fidelity simulation model of the environment is necessary [7]. Such a model should include all relevant effects that can affect the device under test (DUT) operation. It should also, with the use of the test bed, reproduce the two-way interaction between the modeled environment and the tested system. The model’s ability to work in the real-time domain and at a frequency high enough not to affect the tested system’s dynamics is another important aspect.

Such models have already been developed and described for automotive, marine, and aerospace systems [8,9,10]. Their use is also noticeable in the rocket industry for glide experiments and military grade missiles [6,11,12]. Development tools, adapted for numerical modeling, simulation, and real-time systems integration, are also available. However, no off-the-shelf solutions, which could be applied without modification to a suborbital rocket flight analysis, were found. Therefore, it was necessary to develop in-house tools, adapted for the described case.

It was decided that the model should be compatible with the hardware and software used for the ILR-33 AMBER rocket development. Matlab/Simulink, version R2023a^®^ software and the Speedgoat^®^ real-time computer were then used. A model based on physical equations was designed, refined by measurement data and experimental results. Aerodynamic characteristics, engine thrust, and time-varying mass parameters were used to simulate the flights dynamics accurately, therefore simulating the system external loads. Significant phenomena causing flight trajectory perturbations were also modeled. To simulate the control system input signals correctly, the model takes into account selected on-board systems, such as the inertial measurement unit, GNSS receivers, and the on-board computer.

Development of communication interfaces between the DUT and model outputs and inputs was one of the crucial tasks. A modular design of the model permitted us to perform this task in a way that does not affect other system elements. The adopted modular philosophy also permits us to (potentially) substitute the tested device with its digital equivalent for comparison tests.

**Figure 1:**
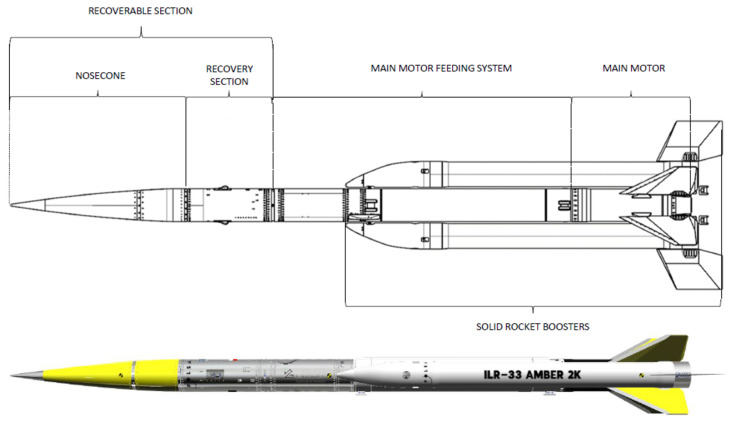
The ILR-33 AMBER 2K outline.

The ILR-33 AMBER is a suborbital platform enabling technology tests and microgravity investigations to be performed at altitudes up to approximately 100 km (the Kármán line). It has been developed entirely in-house by Ł–ILOT. The ILR-33 AMBER is a two-stage rocket (parallel stages) propelled by a hybrid main motor (high-test peroxide (HTP) 98% + high-density polyethylene (HDPE)) and two solid rocket boosters (SRB-s). The recoverable section, despite the elements outlined in Figure 1, can also include a control system if required by mission needs.

**Figure 2:**
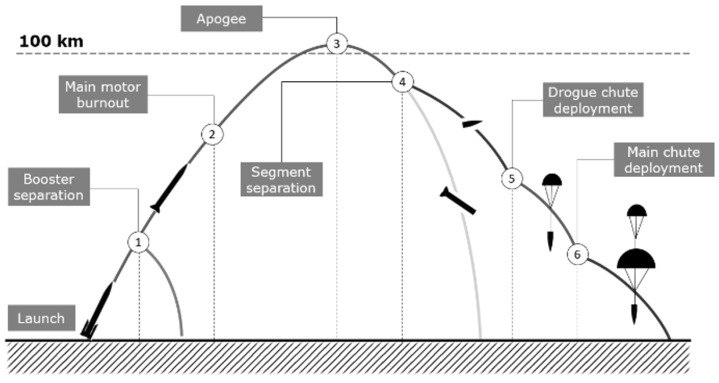
The ILR-33 AMBER 2K mission profile.

The typical mission profile is shown in Figure 2. The rocket is launched with SRB-s propelling the vehicle. After SRB-s burnout, these are separated from the core (1), and the main stage continues the ascent, with the hybrid motor being ignited (at any time, as required by the specific mission) and firing until the programmed burnout (2). Once the rocket reaches the apogee (3), the recovery phase begins with the stages separation. The recoverable section enters flat spin motion to decelerate significantly prior to parachute operation (4). At the programmed altitudes, the pilot (drogue) parachute (5) and main parachute open (6) to ensure a soft touchdown.

Having combined both ILR-33 versions, it was flown four times from Poland; the maiden flight took place in 2017 at Drawsko Training Center (apogee of 15 km). There were two flights in 2019, the first at Drawsko (10 km) and the second at the Air Force Training Center (AFTC) in Ustka (23 km), respectively. The latest attempts allowed us to maturize the platform (2022, Ustka-8 km) and demonstrate the rocket’s operability in high-altitude conditions (apogee of 101 km, Norway, 2024).

## 2. Test Stand

**Figure 3:**
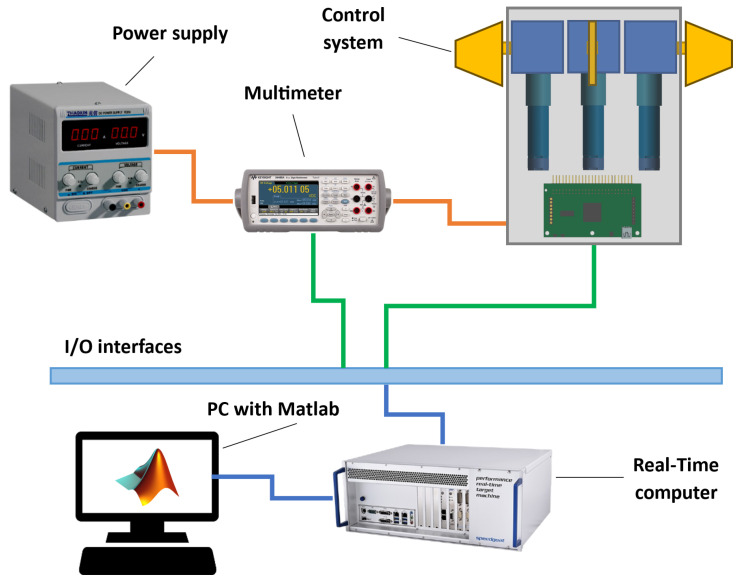
Scheme of the HiL test stand elements.

**Figure 4:**
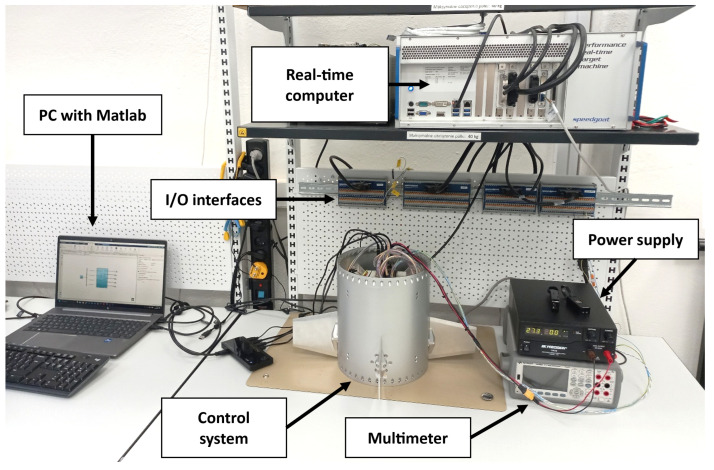
Test stand for the ILR-33 AMBER rocket’s control system.

The HiL test stand for the control system of the ILR-33 AMBER rocket serves as an integrated testing environment, in which a real component of the control system is tested under conditions as close to reality as possible. The structure of the test stand, presented in Figure 3, includes the key elements necessary for conducting reliable simulations and analyses. The real test stand used during HiL tests is shown in Figure 4.

The main components of the HiL test stand include:

- Laboratory power supply—Provides stable supply voltage to the DUT.
- Multimeter—Used to monitor voltage and current between the power supply the DUT.
- Control system DUT—Physical rocket component responsible for executing canards deflection, including motion controllers and servomechanisms.
- Real-time computer—Responsible for executing the simulation model in real time and handling communication with the DUT and the multimeter.
- PC workstation—Equipped with Matlab/Simulink^®^ software, used to monitor and analyze simulation results. It also allows for editing model parameters and visualizing measurement data.
- Input/Output interfaces—Used to connect the control system and the multimeter with the real-time computer.

## 3. Simulation Model

### 3.1. Structure

**Figure 5:**
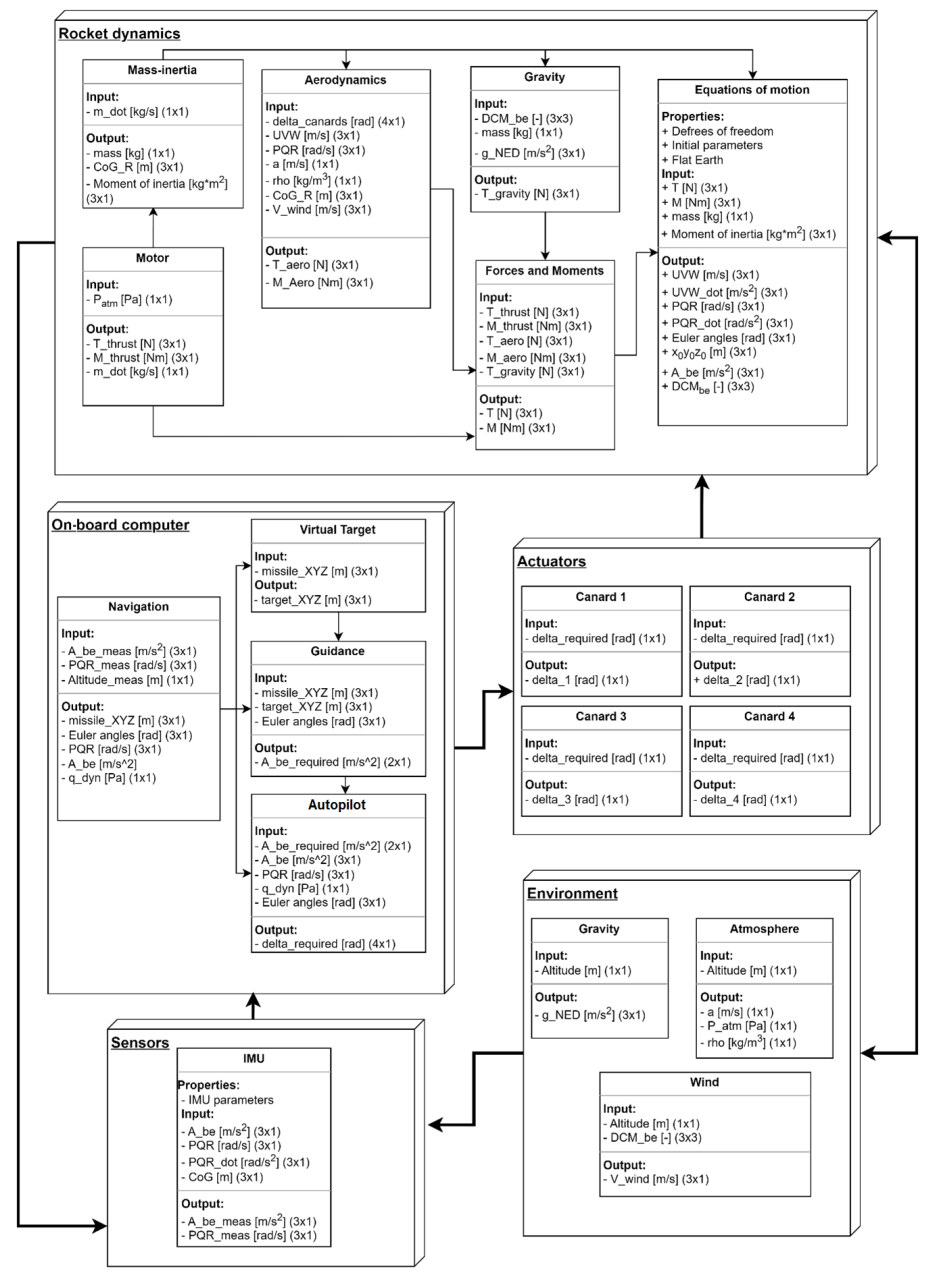
Diagram of the simulation model.

The simulation model structure consists of five main modules. These modules are interconnected, which is shown in Figure 5. The data flow between modules includes both the inputs and outputs of each unit as well as their inter-dependencies. Model modules are:

- **Rocket dynamics**—Includes 6DOF equations of motion, aerodynamics and thrust forces models, and representation of the rocket’s variable mass and inertia properties. It calculates the rocket’s current motion state based on external and internal forces and moments.
- **Sensors**—Simulates the operation of on-board inertial sensors (IMUs), GNSS receivers, and pressure sensors.
- **On-board computer**—Responsible for executing navigation, guidance, and control algorithms; processing sensor data; and generating control commands for the actuators.
- **Actuators**—In the case of MiL (Model-in-the-Loop) tests, it includes motion controllers and servomechanism models, built on the basis of methods described in [13]. In HiL tests, it is limited to a block that generates output signals for the DUT and decodes input signals from it.
- **Environment**—Models’ external conditions such as gravity, atmospheric properties, and wind, taking into account varying environmental parameters related to flight dynamics.

The model allows the simulation model to reproduce the rocket’s flight dynamics and the interactions between the control, guidance, and flight dynamics systems.

The modular design is such that components can be easily modified or replaced, if necessary. It facilitates the adaptation to changing requirements or specific test scenarios, ensures flexibility in development, and enables the rapid changes implementation without the need for simulation framework redesigning. Furthermore, the modular architecture facilitates the verification of individual components, promoting independent testing and helping to identify potential calculation errors. The model can also be easily expanded with additional modules, such as new types of sensors or actuators. Such flexibility is crucial in the context of research on emerging rocket technologies, which often require regular updates and adaptations during research and development progression.

### 3.2. Assumptions

The main model assumptions and simplifications are listed below. Certain phenomena were intentionally omitted to improve computational efficiency and adapt the model for real-time testing. These simplifications do not significantly affect the accuracy of rocket behavior in the scenarios analyzed:

- ILR-33 AMBER-guided configuration is modeled. It differs slightly from the unguided version, flown in 2024 [14], in terms of total length, mass-inertia characteristics, and aerodynamics. It is then expected that the flight trajectories of both versions can differ.
- Only the part of the flight after boosters separation is modeled. Initial conditions for the simulation (separation time, velocities, positions) are predicted based on a full flight model developed by Ł-ILOT for the ILR-33 AMBER rocket and introduced as constant inputs.
- The rocket is considered as a rigid body (with deflective rigid canards),
- Mass-inertia characteristics are variable in time- and event-dependent modes (rocket motors operation).
- Mass-inertia characteristics are symmetrical in relation to XY and XZ planes. Principal moments of inertia about the Y and Z axes are equal: $I_{y}=I_{z}=I$.
- The sensors are ideal (no dynamics, delays, or measurement errors).
- No thrust force misalignment, resulting in transverse thrust forces and moments.
- A flat Earth model is used, as the impact point distance from the launch position is much lower than the Earth curvature radius. The impact point and apogee accuracy will be then sufficiently accurately reproduced for HiL testing.
- Magnus force is neglected, as its influence on the rocket’s trajectory is marginal compared with dominant classic aerodynamic and gravitational forces.
- Coriolis force is neglected due to the short duration of flight and the rocket’s limited range.
- Jet-damping effect is omitted, as its impact on flight stability is much lower than aerodynamics and canards deflection.

### 3.3. Frames of Reference

The dynamics model is based on rectangular right-handed coordinate systems, according to the convention proposed by Kowaleczko [15]. Several coordinate systems were used during model development:

- $O_{g}x_{g}y_{g}z_{g}$—Topographic coordinate system with origin on the Earth’s surface, where axis $z_{g}$ points towards the center of the Earth and the $O_{g}x_{g}y_{g}$ plane is tangential to the Earth’s surface.
- $Ox_{g}y_{g}z_{g}$—Coordinate system with axes parallel to $O_{g}x_{g}y_{g}z_{g}$, with its origin at the rocket’s center of mass.
- Oxyz—Body-fixed coordinate system attached to the rocket.
- $Ox_{a}y_{a}z_{a}$—Aerodynamic coordinate system associated with the airflow around the rocket.
- $Ox_{ab}y_{ab}z_{ab}$—Aerodynamic coordinate system associated with the body frame (rocket).
- $Ox_{c}y_{c}z_{c}$—Coordinate system associated with the rocket’s control surfaces.

**Figure 6:**
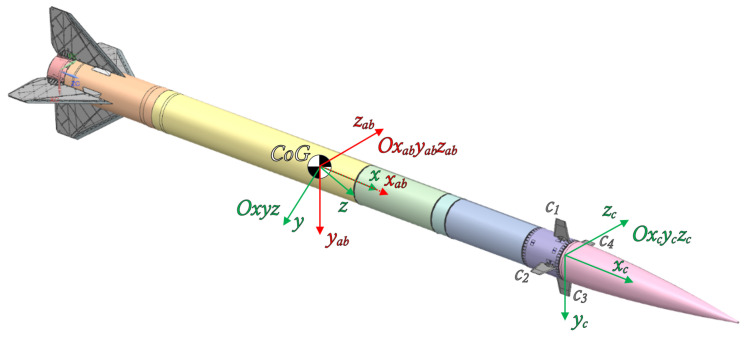
Rocket coordinate systems.

**Figure 7:**
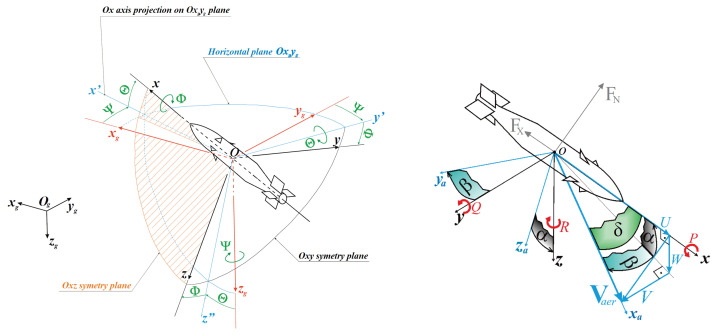
Aerodynamics and flight dynamics coordinate systems and relationships between them [15].

The graphical representation of the coordinate systems and relations between them are shown in Figure 6 and Figure 7.

### 3.4. Rocket Dynamics

The rocket dynamics block is responsible for simulating the guided rocket flight in a specified environment. The second stage of the ILR-33 AMBER rocket is equipped with aerodynamic control surfaces in a canard configuration and a hybrid rocket motor as the main propulsion.

The 6DOF dynamics model was developed based on the equations of motion presented by Kowaleczko [15]. It represents a mathematical and physical description of the dynamics of a variable-mass object, taking into account the main forces acting on it during flight. Three main external force sources were considered: aerodynamics, gravity, and thrust. Based on assumptions mentioned above, equations of motions were formulated:

(1)U˙=Fx+m(RV−QW)m(2)V˙=Fy+m(PW−RU)m(3)W˙=Fz+m(QU−PV)m(4)P˙=MxIx(5)Q˙=My+PR(I−Ix)I(6)R˙=Mz+PQ(Ix−I)I(7)Φ˙=P+(QsinΦ+RcosΦ)tanΘ(8)Θ˙=QcosΦ−RsinΦ(9)Ψ˙=QsinΦ+RcosΦcosΘ(10)x˙=UcosΨcosΘ+V(cosΨsinΘsinΦ−sinΨcosΦ)+W(cosΨsinΘcosΦ+sinΨsinΦ)(11)y˙=UsinΨcosΘ+V(sinΨsinΘsinΦ+cosΨcosΦ)+W(sinΨsinΘcosΦ−cosΨsinΦ)(12)z˙=−UsinΘ+VcosΘsinΦ+WcosΘcosΦ

where:

- ${\left[x,y,z\right]}^{T}$—Position of the object in the topographic coordinate frame [m].
- $V={\left[U,V,W\right]}^{T}$—Linear velocity vector of the object in the body frame [m/s].
- $\Omega ={\left[P,Q,R\right]}^{T}$—Angular velocity vector of the object in the body frame [rad/s].
- $F={\left[F_{x},F_{y},F_{z}\right]}^{T}$—Resultant external force acting on the object in the body frame [N].
- $M={\left[M_{x},M_{y},M_{z}\right]}^{T}$—Resultant moment of external forces acting on the object in the body frame [Nm].
- *m*—Mass of the object [kg].
- $I_{x}$—Moment of inertia about the longitudinal axis Ox [kg·$m^{2}$].
- *I*—Moment of inertia about axes Oy and Oz [kg·$m^{2}$].
- ${\left[\Phi ,\Theta ,\Psi \right]}^{T}$—Euler angles representing roll, pitch, and yaw with respect to the inertial frame [rad].

The mass-inertia properties module outputs the current mass, center of gravity position, and moments of inertia. The rocket’s mass, center of gravity, and moments of inertia are a function of the motor’s mass flow rate in time. These parameters are calculated using predefined tables specific to the ILR-33 AMBER rocket, determined on CAD model measurements and main motor experimental results.

The thrust model is based on relations defining the exhaust gas velocity and mass flow rate as functions of time. The thrust includes contributions from both the exhaust gas momentum and the pressure difference across the nozzle exit area. Mathematically, this is expressed as follows:

(13) $$T=\dot{m}\left(t\right)V_{e}\left(t\right)+\left(p_{e}\left(t\right)-p_{0}\right)A_{e}\left(t\right)$$

where:

- *T*—Thrust [N].
- $\dot{m}\left(t\right)$—Mass flow rate as a function of time [kg/s].
- $V_{e}\left(t\right)$—Exhaust gas velocity as a function of time [m/s].
- $p_{e}$—Exhaust pressure as a function of time [Pa].
- $p_{0}$—Ambient atmospheric pressure as function of altitude [Pa] (according to the atmosphere model, see chapter 3.6).
- $A_{e}\left(t\right)$—Nozzle exit area as a function of time [m^2^].

These parameters are determined based on ILR-33 AMBER main motor characteristic tables. As mentioned, the motor is assumed to have zero offset (no thrust misalignment), implying no lateral forces along the rocket’s longitudinal axis. Consequently, thrust-induced moments are also neglected, simplifying the system dynamics and allowing for more efficient numerical integration of the motion equations.

### 3.5. Aerodynamics

**Figure 8:**
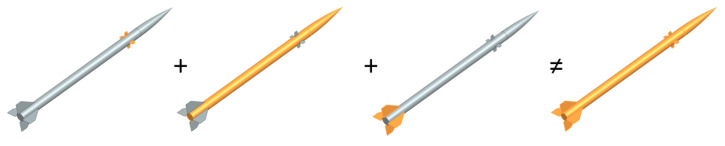
Graphical breakdown of the rocket and explanation of aerodynamic interference. The sum of isolated (highlighted) components does not result in the same characteristics as the whole-body assembly of those components. Source: own work. Siemens NX software, version 2206 was used.

Among several approaches to aerodynamic modeling, simple yet efficient semi-empirical formulas were selected to learn the behavior of the control system. It allows one to model various geometries for rockets with no need for precise but time-consuming calculations (offered by CFD analysis). The aerodynamic model (Figure 8) consists of four control surfaces (canards), an axis-symmetric core (composed of a parabolic head and a cylindrical body), and four stabilizers in the aft of the body (fins).

**Figure 9:**
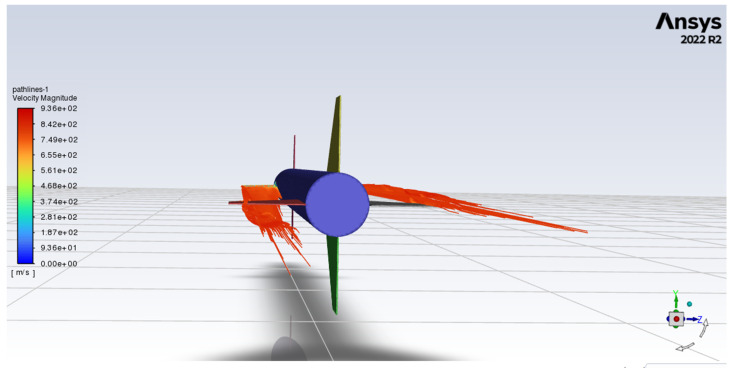
Downwash visualization in ANSYS Fluent simulation of the ILR-33 AMBER analysis. Source: own work. Mach = 2.5, angle of attack = 0 deg, canards deflection = ±5 deg.

Concerning limitations, the model assumes linear dependency on the angles (of attack, sideslip, canard deflection) and no lift generation by the cylindrical body. Those limitations are valid until the rocket does not enter angles of attack or sideslip over magnitudes of 8–10 deg. Furthermore, aerodynamic force and moment coefficient formulas usually refer to isolated elements, and total values comprise the superposition of the aforementioned components. Despite the component and superposition approach, one of the significant interference (Figure 8) features was modeled, namely body-to-surfaces (increased lift on the surfaces due to presence of the body). There was no surface-to-body nor canards-to-fins interference components modeled. That leads to underprediction of the real rocket’s angles in control maneuvers. It is caused by the fact that, in reality, where interference is included, the canards’ deflection affects fins, so these start generating normal force in the direction opposite to the canards’ force (due to shedding vortices). As a result, the fins start magnifying the pitching or yawing moment until the rocket enters higher angles of attack, when fins generate higher normal force back with the same direction as the canards’ normal force direction. Figure 9 presents a case for ILR-33 AMBER, where two canards are deflected: the left canard (C2, wrt Figure 6) is deflected, such that the flow deflects downwards downstream; and the right canard (C4) in the opposite direction. Thus, the rocket shall rotate clockwise. However, such a deflection configuration leads to induced angles of attack on fins resulting in opposite force and thus opposite rolling moment (counterclockwise).

The aerodynamic force (in [N]) and moment (in [Nm]) are defined as follows (in frame $Ox_{ab}y_{ab}z_{ab}$):

(14) $$\begin{array}{cc} F_{ab} & =qS_{\mathrm{ref}}\left[\begin{array}{c} C_{x}\\ C_{y}\\ C_{z} \end{array}\right], \end{array}$$

(15) $$\begin{array}{cc} M_{ab} & =qS_{\mathrm{ref}}L_{\mathrm{ref}}\left[\begin{array}{c} C_{mx}\\ C_{my}\\ C_{mz} \end{array}\right] \end{array}$$

where:

- *q* is the dynamic pressure varying during the flight [Pa].
- **$S_{\mathrm{ref}}$** is the reference area, equal to $0.0415\;m^{2}$.
- **$L_{\mathrm{ref}}$** is the reference length, equal to 0.23m.

The moment refers to the rocket’s center of gravity, being the origin of the used coordinate system. The normal force can be distinguished in two planes, determined by angles of attack (angle in the XY plane of the aerodynamic coordinate system) and sideslip (angle in the XZ plane), respectively. For fins and canards, two formulas were used to determine normal force coefficients, dependent on Mach number [16]. For Mach ≤ 0.8:

(16) $${\left(\frac{\partial C_{\mathrm{normal}}}{\partial \mathrm{ANGLE}}\right)}_{\left(\alpha ,\beta ,\delta \right)}^{\mathrm{pair}\;\mathrm{of}\;\mathrm{surfaces}}=\frac{1.84\pi \lambda_{\mathrm{surfaces}}}{2.4+\lambda_{\mathrm{surfaces}}}\cdot \frac{1}{\sqrt{1-{\mathrm{Mach}}^{2}}}\cdot \frac{S_{\mathrm{surfaces}}}{S_{\mathrm{ref}}}\cdot K_{b\text{-}s}\left(\alpha ,\beta ,\delta \right)$$

For Mach ≥ 1.15:

(17) $${\left(\frac{\partial C_{\mathrm{normal}}}{\partial \mathrm{ANGLE}}\right)}_{\left(\alpha ,\beta ,\delta \right)}^{\mathrm{pair}\;\mathrm{of}\;\mathrm{surfaces}}=\frac{4}{\sqrt{{\mathrm{Mach}}^{2}-1}}\left(1-\frac{1}{2\lambda_{\mathrm{surfaces}}\sqrt{{\mathrm{Mach}}^{2}-1}}\right)\cdot \frac{S_{\mathrm{surfaces}}}{S_{\mathrm{ref}}}\cdot K_{b\text{-}s}\left(\alpha ,\beta ,\delta \right)$$

where:

- **α** is the angle of attack, in rad.
- **β** is the sideslip angle, in rad.
- **δ** is the canard’s angle of deflection, in rad.
- **$S_{\mathrm{surfaces}}$** is the lift area (planform area) of a pair of the surfaces (canards or fins), $m^{2}$.
- $\lambda_{\mathrm{surfaces}}$ is the aspect ratio for a pair of surfaces (canards or fins), -.
- *K*_**b-s**_ is the body-to-surfaces (canards or fins) interference coefficient, -.

For Mach numbers between 0.8 and 1.15, coefficients were interpolated. The fins’ center of pressure along the rocket’s longitudinal chord was modeled by [17].

For the head, an approximation from [18] for a parabolic head with an elongation of five was selected to calculate normal force derivatives. The center of pressure was assumed to be located at the midpoint of the body length [17].

All interference factors are obtained from [17]. Based on that, the interference factors differ depending on whether the entire body is exposed to the angle of attack or there is just the deflection of canards with no angle of attack or sideslip. For instance, $K_{b\text{-}s}$ for δ is equal to one, while for α or β, values are greater than one. For canards, to obtain the total normal force coefficient, superposition of normal force-to-deflection and normal force-to-angle of attack/sideslip is used.

The normal force for both Y and Z directions is calculated as the sum of normal force originating from non-zero angles and damping normal force from angular velocity during dynamic maneuver:

(18) $$\begin{array}{cc} C_{y} & =C_{y}\left(\alpha ,\delta ,Mach\right)+C_{y}^{\mathrm{damp}}\left(R,Mach\right) \end{array}$$

(19) $$\begin{array}{cc} C_{z} & =C_{z}\left(\beta ,\delta ,Mach\right)+C_{z}^{\mathrm{damp}}\left(Q,Mach\right) \end{array}$$

(20)Cy(α,δ,Mach)=α∂Cnormalhead∂α+∂Cnormalfins∂α+∂Cnormalcanards∂α+δ∂Cnormalcanards∂δ(21)Cydamp(R,Mach)=Rv∞∂Cnormalhead∂αx¯head+∂Cnormalfins∂αx¯fins+∂Cnormalcanards∂αx¯canards

(22)Cz(β,δ,Mach)=β∂Cnormalhead∂β+∂Cnormalfins∂β+∂Cnormalcanards∂β+δ∂Cnormalcanards∂δ(23)Czdamp(Q,Mach)=Qv∞∂Cnormalhead∂βx¯head+∂Cnormalfins∂βx¯fins+∂Cnormalcanards∂βx¯canards

where:

- **$v_{\infty }$**—Free-stream velocity, in m/s.
- **${\bar{x}}_{\mathrm{head}}$**—Distance from the head’s center of pressure to the origin of the aerodynamic coordinate system, in m.
- **${\bar{x}}_{\mathrm{fins}}$**—Distance from the fins’ center of pressure to the origin of the aerodynamic coordinate system, in m.
- **${\bar{x}}_{\mathrm{canards}}$**—Distance from the canards’ center of pressure to the origin of the aerodynamic coordinate system, in m.

The axial force coefficient was determined using semi-empirical formulas from [19]. Due to low angles of attack or sideslip, the axial force is Mach-dependent only:

(24) $$C_{x}\left(Mach\right)=C_{x}^{\mathrm{friction}}+C_{x}^{\mathrm{base}}+C_{x}^{\mathrm{wave}}$$

where:

- **Friction** is present throughout the flight (function of wetted surface and Reynolds number).
- **Base drag** appears after propulsion ends (function of Mach and aft diameter).
- **Wave drag** appears for Mach 1 or higher (function of head elongation).

Aerodynamic moment components are calculated as follows:

(25) $$\begin{array}{cc} C_{my} & =C_{my}\left(\beta ,\delta ,Mach\right)+C_{my}^{\mathrm{damp}}\left(Q,M\right) \end{array}$$

(26) $$\begin{array}{cc} C_{mz} & =C_{mz}\left(\alpha ,\delta ,Mach\right)+C_{mz}^{\mathrm{damp}}\left(R,M\right) \end{array}$$

(27)Cmy(β,δ,Mach)=β∂Cnormalhead∂βx¯head+∂Cnormalfins∂βx¯fins+∂Cnormalcanards∂βx¯canards+δ∂Cnormalcanards∂δx¯canards(28)Cmydamp(Q,Mach)=Qv∞∂Cnormalhead∂βx¯head2+∂Cnormalfins∂βx¯fins2+∂Cnormalcanards∂βx¯canards2(29)Cmz(α,δ,Mach)=α∂Cnormalhead∂αx¯head+∂Cnormalfins∂αx¯fins+∂Cnormalcanards∂αx¯canards+δ∂Cnormalcanards∂δx¯canards(30)Cmzdamp(R,Mach)=Rv∞∂Cnormalhead∂αx¯head2+∂Cnormalfins∂αx¯fins2+∂Cnormalcanards∂αx¯canards2

(31) $$\begin{array}{cc} C_{mx} & =C_{mx}^{\mathrm{induced}\;\mathrm{external}}+C_{mx}\left(\delta ,Mach\right)+C_{mx}^{\mathrm{damp}}\left(P,Mach\right) \end{array}$$

where:

- $C_{mx}^{\mathrm{induced}\;\mathrm{external}}$—Any unintended external rolling moment occurring during flight, in Nm.
- **${\bar{y}}_{\mathrm{fins}}$**—Distance from the fins’ center of pressure to the origin of the aerodynamic coordinate system in the *y* direction, in m.
- **${\bar{z}}_{\mathrm{fins}}$**—Distance from the fins’ center of pressure to the origin of the aerodynamic coordinate system in the z direction, in m.
- **${\bar{y}}_{\mathrm{canards}}$**—Distance from the canards’ center of pressure to the origin of the aerodynamic coordinate system in the y direction, in m.
- **${\bar{z}}_{\mathrm{canards}}$**—Distance from the canards’ center of pressure to the origin of the aerodynamic coordinate system in the z direction, in m.

(32)Cmx(δ,Mach)=∑i=1n=4δi·12∂Cnormalcanards∂δy¯canards∨z¯canards(33)Cmxdamp(P,Mach)=Pv∞∂Cnormalfins∂αz¯fins2+∂Cnormalfins∂βy¯fins2+∂Cnormalcanards∂αz¯canards2+∂Cnormalcanards∂βy¯canards2

The static margin is calculated by the model based on pitch and yaw moments. It is assumed that all normal forces and moments are linearly dependent on the angles of attack and sideslip (valid for low angles of attack, magnitude of 5 deg), so the rocket’s center of pressure depends on Mach number only. Center of pressure is then calculated by dividing moments by normal forces values in corresponding axes.

### 3.6. Environment

The environment module consists of three key components: the gravity model, the atmospheric model, and the wind model. The inputs to this module include the altitude above ground level and the geographical latitude of the object. Due to the flat Earth assumption adopted in the simulation, the latitude remains constant and corresponds to the location of the simulated rocket launch site. This parameter affects the gravitational acceleration value in the model only.

The gravity model is based on the ISO 2533 standard [20], which defines the dependence of gravitational acceleration on altitude above sea level. Although this standard also provides other atmospheric parameters, the key aspect for gravity modeling is the variation of Earth’s gravitational acceleration with respect to altitude and geographical latitude. The inputs to the model are the object’s latitude and altitude above the Earth’s surface. The output is the gravitational acceleration that reflects changes due to both altitude and geographic position.

The atmosphere model is based on the COESA standard implemented by MathWorks [21]. Based on the object’s altitude, the model calculates the air temperature, pressure, density, and speed of sound. The model is constrained by a minimum and maximum geopotential altitude, which must fall within the range of 0 to 84,852 m. At this stage, this altitude range is assumed to be sufficient for the purposes of HiL testing of the control system. However, future modifications to the atmospheric model are possible, if required.

The environment module also accounts for the influence of the wind on the rocket’s trajectory. The wind model is implemented using a table of values that defines the wind speed and azimuth as functions of altitude. This enables a realistic simulation of aerodynamic effects caused by atmospheric flows on the rocket’s flight dynamics.

### 3.7. On-Board Computer and Sensors

The on-board computer has a significant impact on the flight trajectory shape, as it integrates sensors signal processing, navigation, guidance, and autopilot algorithms. Its primary task is to process measurement data from on-board sensors in order to determine the current state of the rocket, compute the trajectory, and generate control signals. The computer consists of four main modules:

- **State observer**—Responsible for navigation and estimation of the rocket’s motion parameters such as position, velocity, and spatial orientation.
- **Virtual target generator**—Generates the target point for the guidance law, which will result in an appropriate impact point.
- **Guidance law**—Generates the required control accelerations based on the rocket’s current position and orientation.
- **Autopilot**—Transforms the required accelerations into appropriate control commands for the rocket’s aerodynamic system, ensuring stabilization and trajectory tracking during flight.

The **state observer** acts as the rocket’s navigation system, estimating its position and orientation based on measurements from IMU. The inputs to the observer include the rocket’s linear acceleration, angular velocity, and initial conditions of position and spatial orientation. Based on these inputs, the observer computes the rocket’s actual position relative to the Earth’s surface and spatial orientation with respect to a reference frame. The outputs of the state observer serve as critical inputs for the virtual target generator, guidance, and autopilot modules.

During the trajectory computation, the concept of a **virtual target generation** is used, allowing control to be performed during the ascent phase. The virtual target is defined as a point in space whose position ensures the maintenance of the desired trajectory, specifically the required LOS inclination angle.

The position of the virtual target $P_{t}=\left(x_{t},\;y_{t},\;z_{t}\right)$ depends on the current rocket position $P_{r}=\left(x_{r},\;y_{r},\;z_{r}\right)$, the nominal impact point $P_{f}=\left(x_{f},\;y_{f},\;0\right)$, and the nominal LOS inclination angle $\theta_{LOS}$. The altitude of the virtual target $z_{t}$ is assumed constant and mission-specific. The $x_{t}$ and $y_{t}$ coordinates are updated on each simulation step following the algorithm below:

The $x_{t}$ coordinate of the virtual target in the reference frame $O_{g}x_{g}y_{g}z_{g}$ is determined as follows:

(34) $$x_{t}=x_{r}+\sqrt{d^{2}-{\left(y_{r}-y_{t}\right)}^{2}}$$

where:

(35) $$d=\frac{z_{r}-z_{t}}{\tan(\theta_{LOS})}$$

This geometric relationship ensures that the nominal LOS inclination with respect to the Earth’s surface is maintained.

**Figure 10:**
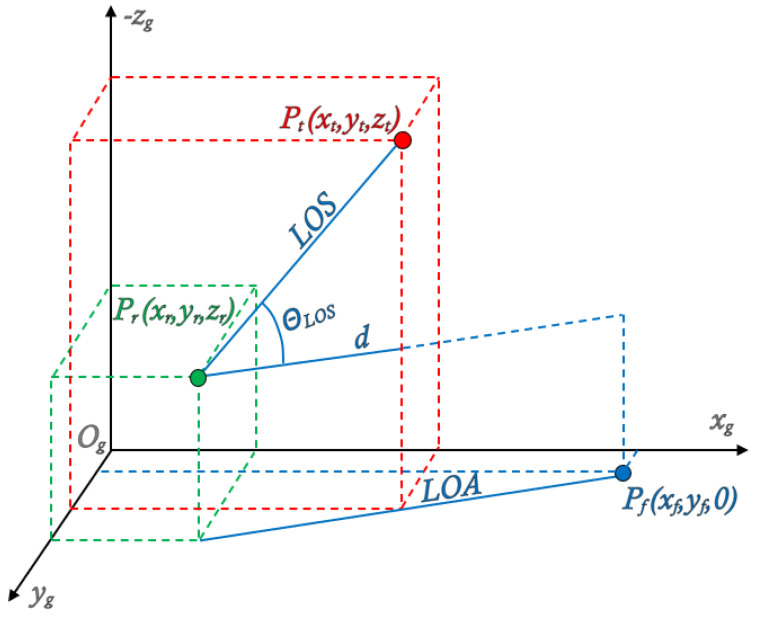
Guidance scheme.

The $y_{t}$ coordinate of the virtual target is computed as:

(36) $$y_{t}=y_{r}\cdot \frac{x_{f}-x_{t}}{x_{f}-x_{r}}$$

which ensures that the virtual target lies on the line of attack (LOA), defined by the current position of the rocket and the nominal impact point. This definition allows the application of proportional navigation to control the rocket during ascent, ensuring trajectory conformity and meeting impact location requirements. A graphical representation of the virtual target generation principle is shown in Figure 10.

The **guidance algorithm** is based on the principle of proportional navigation [7,22]. The acceleration command is calculated as:

(37) $${\vec{a}}_{N}={\vec{\omega }}_{LOS}\times {\vec{V}}_{r}\cdot N$$

where:

- ${\vec{a}}_{N}$—The acceleration command vector [m/$s^{2}$].
- *N*—The navigation constant [-].
- ${\vec{V}}_{r}$—The relative velocity between the rocket and the target [m/s].
- ${\vec{\omega }}_{LOS}$—The line of sight (LOS) angular velocity vector [rad/s].

The **autopilot** module operates in three control channels: roll, pitch, and yaw. Each channel is responsible for controlling the rocket’s motion around its corresponding axis. The roll channel uses a proportional controller in relation to the angular velocity *P*. The pitch and yaw channels are using PI controllers in relation to the linear acceleration *a*. An additional damping factor is introduced in those control loops, respective to angular velocities *Q* and *R*, to reduce unwanted oscillations and enhance the rocket’s dynamic stability. Total deflections in each control channel are then calculated based on formulas derived from [7] (*r* index for roll, *p* for pitch, *y* for yaw):

(38)δr=−P·K1r(39)δp=ep·(K1p+1sK2p)+Q·K3p+K4p·sK5p+s(40)δy=ey·(K1y+1sK2y)+R·K3y+K4y·sK5y+s

In these equations, all instances of *K* are gains, whose values are scheduled based on dynamic pressure *q*. This approach permits us to tune the autopilot with desired stability margins for a wide range of flight conditions. $e_{p}$ and $e_{y}$ are differences between acceleration command values and measurements in body frame and are defined by formulas:

(41)ep=azN+az(42)ey=ayN+ay

In the second step, $\delta_{r}$, $\delta_{p}$, and $\delta_{y}$ are combined to obtain total deflections for each canard. Conventional conversion logic is described in [23] and adapted for the canard configuration shown in Figure 6:

(43)δ1=δp−δr(44)δ2=−δy−δr(45)δ3=−δp−δr(46)δ4=δy−δr

**Figure 11:**
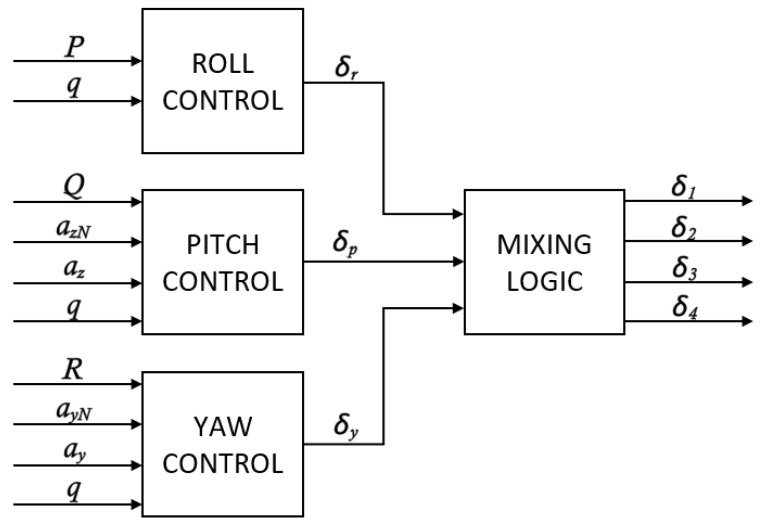
General autopilot diagram.

Moreover, deflection saturation limits are set for each channel and each canard at −10 to +10 degrees. A general autopilot diagram is shown in Figure 11.

The input to the on-board computer model consists of data from the sensor module. In this case, only the IMU model is used. The simulation of the IMU is based on the “Three-axis Inertial Measurement Unit” model developed by MathWorks [24] in “ideal sensor” mode. This model simulates a three-axis accelerometer and a three-axis gyroscope.

## 4. Hardware and Communication

Hardware-in-the-Loop simulation enables testing of the designed hardware and communication interfaces between components. This approach has many benefits. First, the operating conditions closely resemble real-world scenarios, allowing real-time detection of errors during testing. Furthermore, HiL simulations help reduce costs as they do not require physical prototypes or flight tests. In addition, error detection and debugging are much faster and easier when designing such systems.

For this purpose, the Speedgoat real-time computer was used, on which the dynamics of the rocket and environment were simulated. The simulation model is described in Section 3. Running the simulation on a real-time computer is possible thanks to the Matlab^®^ tool, which supports the code generation from Simulink^®^ models and automatic deployment to external hardware such as the Speedgoat^®^ computer. The Speedgoat^®^ computer offers various types of input/output interface modules, allowing us to test analog and digital signals, as well as communication protocols such as ARINC, CAN, UDP, and many others [25]. In the tested system, the CAN [26] protocol was used to send the commanded position of the motor shaft to its drives.

Figure 3 shows all the components of the test stand. Simulink^®^-generated code is transferred from the PC to the Speedgoat^®^ computer via an Ethernet connection before simulation starts. The Speedgoat^®^ computer uses two different communication input/output interfaces. The first interface is Ethernet, through which the multimeter transmits the current draw of the entire system to the Speedgoat^®^ computer. This is performed by connecting the multimeter in series between the power supply and the HiL system. The second interface connects the control executive system to the Speedgoat^®^ computer using the CAN interface, which allows data to be exchanged between the simulation and motors.

In the HiL setup, we can distinguish three levels: on the top of this hierarchy there is the Speedgoat^®^ computer, responsible for simulating the mission, including the rocket dynamics and its subsystems. The implemented rocket control algorithms send data to motor controllers via the CAN interface with desired deflections of the control surfaces. Based on the data from encoders, which measure the actual shaft position, the control algorithms generate phase current signals to rotate the motor shaft and precisely control the motor windings. Depending on the commutation method, these can be trapezoidal pulses or sinusoidal control signals, providing smoother and more efficient motor operation. Speedgoat^®^ sends appropriate commands to the motor drives according to the CAN communication manual provided by Faulhaber [27].

**Figure 12:**
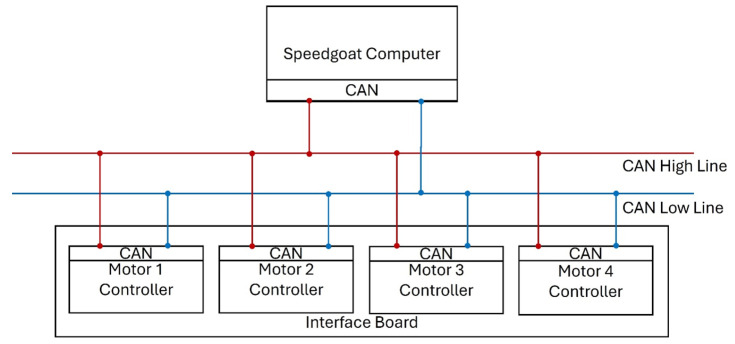
CAN interface architecture.

In the presented configuration, the Speedgoat^®^ computer is a master; the interface board allows for connection with all motors, which are addressed as individual devices with proper identifiers. Motors with controllers are connected to a single interface board, which allows for serial communication between the motors and a real-time computer. Controller area network (CAN) was chosen as the communication protocol. This type of communication is characterized by high reliability, efficiency, and high transmission speed. Moreover, it is a multi-point communication that allows for the exchange of data between multiple devices in the network. The messages can be prioritized, and those with higher priority are transmitted first. Figure 12 presents the interface architecture of the interface used in the described system. All of the components are connected to both the high and low line of the CAN interface. This approach allows all components to send and receive data simultaneously.

HiL simulation is running with a frequency of 250 Hz; this means that the Speedgoat^®^ computer exchanges data with motor controllers 250 times per 1 s. In each iteration, the master (Speedgoat^®^) sends request messages to motors, asking for information about the current position and shaft rotational speed. When the request message is received, the motors reply to the master with the specified message containing the data. Each time any of the components receives a message, it checks its correctness. First, each message contains a CRC calculated based on the message contents. The receiver calculates the CRC and compares it with the one calculated by the sender. Furthermore, the components exchange an ACK (acknowledge) signal with each other in order to confirm that the received message is correct.

## 5. Results and Discussion

**Figure 13:**
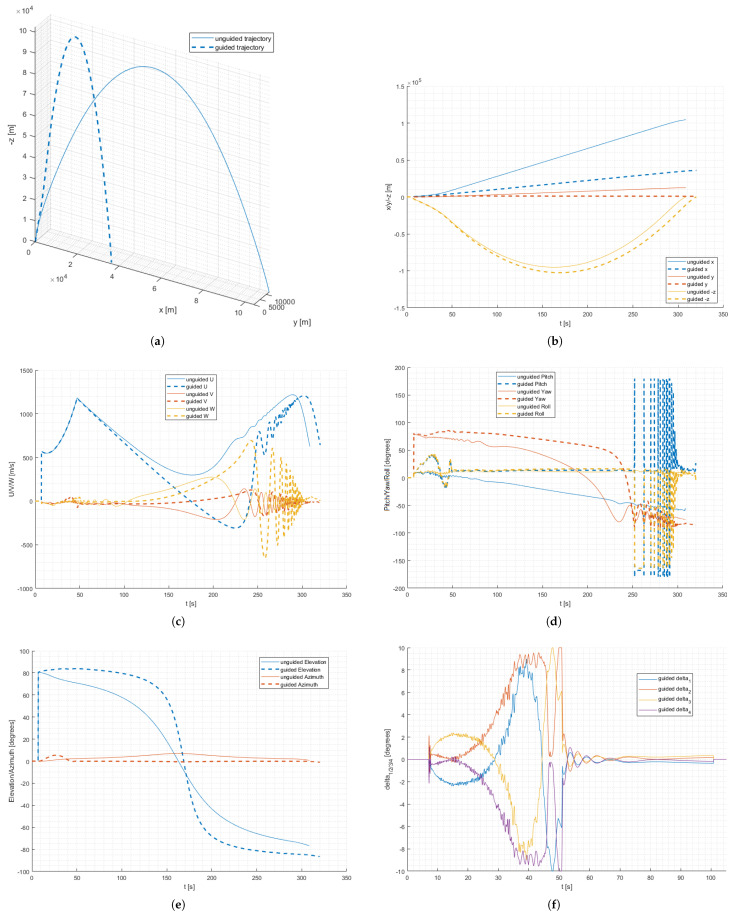
HiL test results: (**a**) 3D flight trajectory. (**b**) xyz position in time. (**c**) UVW velocities in time. (**d**) Pitch, yaw, and roll angles in time. (**e**) Elevation and azimuth angles in time. (**f**) Canards deflections in time.

In order to verify the model, the communication, and the test hardware performance, a set of HiL tests was executed. Two representative flight cases were selected. In the first, the canards are fixed and the rocket follows a ballistic trajectory. In the second case, the on-board computer sends canards deflection commands to the actuators, and the flight trajectory is constantly controlled. Both cases include wind-related disturbances. The algorithm was tuned in a way to reduce the distance between the impact point and the launch site, minimize deviations in the lateral direction, and reduce rockets’ rotation around its longitudinal axis. The obtained results are presented on charts below (Figure 13).

The first trajectory shows a typical flight of an unguided suborbital rocket (Figure 13a). Up to about 47 s, the rocket accelerates due to the main motor thrust. After the main motor shutdown, the rocket continues its flight on an ascending trajectory, gradually reducing speed up to the apogee. An increase in speed occurs again during the descent phase (Figure 13b,c). Oscillations in transverse axes are also clearly visible on the velocity graph, due to increasing aerodynamic forces in lower atmosphere layers. A renewed velocity reduction can be noticed when the aerodynamic drag exceeds the gravitational force. The rocket’s elevation value is higher at the beginning and decreases gradually during the flight (Figure 13d,e). On the azimuth plot, it can be seen that the rocket experiences an Y-axis deviation at the beginning of the flight, probably due to wind gusts. This deviation is not compensated, resulting in an impact point deflection of about 11 km from the initial firing plane. The downrange impact point and flight ceiling are also slightly inferior from the unguided version flown in 2024 [14], which was expected.

In the case of a guided trajectory, a significant change in all flight parameters relative to the uncontrolled trajectory is clearly visible (Figure 13a–d). This is an expected effect and results from the control sequence performed. The elevation is maintained at above 80 degrees for more than 90 s of flight (Figure 13e). This results in a much steeper flight trajectory, slightly higher ceiling, and a closer impact point. It can also be observed that the occurring wind gusts cause a disturbance in the Y-axis. This can be seen in the azimuth plot for the first 50 s of flight. However, the effect of the wind on the trajectory is compensated by the guidance algorithm, resulting in an impact in the vicinity of the initial launch plane. It is also worth noting that the negative velocity of the rocket in the longitudinal axis (“U” value) is not a calculation error but is caused by the angle of attack exceeding 90 degrees. This is possible because of the very low atmosphere density and small aerodynamic forces in the upper parts of the trajectory.

A lot of information can be taken from the canards deflection chart (Figure 13f). It can be seen that these are moving in pairs and that the direction of the deflection is dependent on the current angular rocket orientation. Canards deflections are relatively small (about 2 degrees) up to the 25th second of flight and then gradually increase. It is caused by the decrease in aerodynamic forces resulting from the lowering atmosphere density. Short saturation periods can be seen at about 45 to 52 s of flight. After 55 s of flight, the deflections decrease again as the rocket reaches a required ballistic trajectory, which allows it to reach the desired impact point. Only gentle adjustments at small deflections are generated at this point. After about 100 s, the canards return to the zero position, where they remain for the rest of the flight. In the initial part of the trajectory, high-frequency and low-altitude oscillations in their movement are also visible, presumably to compensate for the effect of the wind on the trajectory. The oscillations do not cause any performance degradation in the flight path compensation algorithm.

## 6. Conclusions

In this paper, a coherent test system developed for a suborbital rocket control system verification was shown. The test stand, the device under test, and the mathematical model description, as well as their mechanical, electrical, and communication interfaces, are described. Then, a set of HIL tests was performed, with the use of representative flight cases for the ILR-33 AMBER rocket. The tests demonstrated that the DUT communicates with the simulated on-board avionics correctly and executes the control sequence. An extended results analysis was performed, including flight trajectories comparison and canards’ behavior observation and identification. All typical phenomena that can occur during such flight, related to the operation of the propulsion unit, aerodynamics, gravity, atmosphere, and wind, were observed and explained. The results obtained are reasonable and coincide with previous simulations and test flights of the ILR-33 AMBER rocket [14,28]. They permit us to collect better understanding on guided suborbital rocket flight.

For the first time, a complete flight trajectory of a suborbital rocket was shown during control system HIL tests. This demonstrates a high utility of the methods used in the suborbital rockets development process. The approach can be applied for any suborbital rocket project, where a high level of reliability is needed from the first launch. This can be particularly helpful for limited-budget projects, where launch failure is unacceptable, or for launches from ranges with very limited impact zone (such simulations allow us to model, for example, control system ability to reduce the impact zone).

To improve model performance and reflect the object’s real behavior, several modifications should be considered in the following activities. Canards-to-fins aerodynamic interference should be investigated thoroughly to predict angle of attack or sideslip angle values more precisely. More complex aerodynamic derivatives may be considered as stated in [29]. More accurate sensors models could also be added to evaluate their impact on the navigation algorithm. Real data from ground and flight tests could be also incorporated in the model, increasing its fidelity. Finally, other subsystems (such as the rocket’s on-board computer) could be integrated into the test setup, becoming new DUTs. It should be noted that the aforementioned improvements should be implemented in a way that does not affect the model computation performance significantly.

## Data Availability

The data presented in this study are available on request from the corresponding author. The data are not publicly available due to privacy restrictions.

## Abbreviations

The following abbreviations are used in this manuscript:

ACK	Acknowledge
ARINC	Aeronautical Radio, Incorporated (protocol)
CAD	Computer-aided design
CAN	Controller Area Network (protocol)
CFD	Computational fluid dynamics
COESA	U.S. Committee on Extension to the Standard Atmosphere
CRC	Cyclic redundancy check
DOF	Degrees of freedom
DUT	Device under test
ECSS	European Cooperatrion for Space Standarization
GNSS	Global Navigation Satellite System
HDPE	High-density polyethylene
HiL	Hardware-in-the-Loop
HTP	High-test peroxide
IMU	Inertial measurement unit
ISO	International Organization for Standardization
LOA	Line of attack
LOS	Line of sight
Ł-ILOT	Łukasiewicz Research Network–Institute of Aviation
MiL	Model-in-the-Loop
PC	Portable computer
SRB	Solid rocket booster
UDP	User Datagram Protocol
